# Endothelial cell TRPA1 activity exacerbates cerebral hemorrhage during severe hypertension

**DOI:** 10.3389/fmolb.2023.1129435

**Published:** 2023-01-30

**Authors:** Michelle N. Sullivan, Pratish Thakore, Vivek Krishnan, Sushma Alphonsa, Wencheng Li, Yumei Feng Earley, Scott Earley

**Affiliations:** ^1^ Oregon National Primate Research Center, Oregon Health and Science University, Beaverton, OR, United States; ^2^ Department of Pharmacology, Center for Molecular and Cellular Signaling in the Cardiovascular System, Reno School of Medicine, University of Nevada, Reno, NV, United States; ^3^ Department of Pathology, Wake Forest University School of Medicine, Winston-Salem, NC, United States; ^4^ Department of Physiology and Cell Biology, Center for Molecular and Cellular Signaling in the Cardiovascular System, Reno School of Medicine, University of Nevada, Reno, NV, United States

**Keywords:** endothelium, TRPA1, stroke, hypertension, hemorrhage

## Abstract

**Introduction:** Hypoxia-induced dilation of cerebral arteries orchestrated by Ca^2+^-permeable transient receptor potential ankyrin 1 (TRPA1) cation channels on endothelial cells is neuroprotective during ischemic stroke, but it is unknown if the channel has a similar impact during hemorrhagic stroke. TRPA1 channels are endogenously activated by lipid peroxide metabolites generated by reactive oxygen species (ROS). Uncontrolled hypertension, a primary risk factor for the development of hemorrhagic stroke, is associated with increased ROS production and oxidative stress. Therefore, we hypothesized that TRPA1 channel activity is increased during hemorrhagic stroke.

**Methods:** Severe, chronic hypertension was induced in control (*Trpa1* ^fl/fl^) and endothelial cell-specific TRPA1 knockout (*Trpa1*-ecKO) mice using a combination of chronic angiotensin II administration, a high-salt diet, and the addition of a nitric oxide synthase inhibitor to drinking water. Blood pressure was measured in awake, freely-moving mice using surgically placed radiotelemetry transmitters. TRPA1-dependent cerebral artery dilation was evaluated with pressure myography, and expression of TRPA1 and NADPH oxidase (NOX) isoforms in arteries from both groups was determined using PCR and Western blotting techniques. In addition, ROS generation capacity was evaluated using a lucigenin assay. Histology was performed to examine intracerebral hemorrhage lesion size and location.

**Results:** All animals became hypertensive, and a majority developed intracerebral hemorrhages or died of unknown causes. Baseline blood pressure and responses to the hypertensive stimulus did not differ between groups. Expression of TRPA1 in cerebral arteries from control mice was not altered after 28 days of treatment, but expression of three NOX isoforms and the capacity for ROS generation was increased in hypertensive animals. NOX-dependent activation of TRPA1 channels dilated cerebral arteries from hypertensive animals to a greater extent compared with controls. The number of intracerebral hemorrhage lesions in hypertensive animals did not differ between control and *Trpa1*-ecKO animals but were significantly smaller in *Trpa1*-ecKO mice. Morbidity and mortality did not differ between groups.

**Discussion:** We conclude that endothelial cell TRPA1 channel activity increases cerebral blood flow during hypertension resulting in increased extravasation of blood during intracerebral hemorrhage events; however, this effect does not impact overall survival. Our data suggest that blocking TRPA1 channels may not be helpful for treating hypertension-associated hemorrhagic stroke in a clinical setting.

## Introduction

Stroke is the second-leading cause of mortality worldwide and the fifth-leading cause of death in the United States (Donkor, 2018; Tsao et al., 2022). Strokes are classified into two categories: ischemic and hemorrhagic. Ischemic stroke is more common and caused by regional interruptions of the blood supply to the brain, a sudden loss of function, and neuronal death. Hemorrhagic stroke results from the rupture of cerebral blood vessels and bleeding into the brain tissue (Bamford et al., 1991). Although hemorrhagic strokes are less common, rates of morbidity and long-term disability following an event are much higher compared with ischemic strokes (Virani et al., 2021). We previously demonstrated that selective knockout of transient receptor potential ankyrin 1 (TRPA1) cation channels from vascular endothelial cells exacerbated neuronal damage following ischemic stroke, and treatment with a TRPA1 agonist improved outcomes (Pires and Earley, 2018). Thus, we concluded that endothelial cell TRPA1 activity is neuroprotective following ischemic stroke. The effects of endothelial cell TRPA1 channels on the incidence or severity of hemorrhagic stroke events have not been previously reported. Here, we investigated how outcomes associated with hypertension-induced hemorrhagic stroke are impacted by cerebral vascular TRPA1 channel activity.

Uncontrolled systemic hypertension is a primary risk factor for hemorrhagic stroke (Hägg-Holmberg et al., 2019). Increased arterial intraluminal pressure enhances smooth muscle cell contractility (Harder et al., 1985; Pratt et al., 2002; Amberg et al., 2003; Bannister et al., 2012) elevating cerebral vascular resistance and augmenting stress on the vascular wall (Hayashi and Naiki, 2009). Greater cerebral perfusion pressure and tangential stress also damages the endothelium over time, weakening the vessel wall to the point of rupture (Izzard et al., 2005; De Ciuceis et al., 2007). Cerebral autoregulation, the process by which cerebral blood flow is maintained during changes in intraluminal pressure, is often impaired during hypertension which contributes to the development of stroke (Strandgaard et al., 1973; Lammie, 2002; Faraco and Iadecola, 2013). Loss of cerebral autoregulation increases perfusion pressure in the capillaries and damages the blood-brain barrier, leading to cerebral edema, inflammation, and neuronal degeneration (Lammie, 2002; Faraco and Iadecola, 2013; Shekhar et al., 2017). Hypertension also enhances the expression of reactive oxygen species (ROS)-generating NADPH oxidase (NOX) enzymes and ROS production in the vasculature (Rajagopalan et al., 1996; Zalba et al., 2001; Landmesser et al., 2003; Adler and Huang, 2004; Kazama et al., 2004; Paravicini et al., 2004; Matsuno et al., 2005). Although ROS are critical for a variety of physiological responses (Knock et al., 2009; Xiao et al., 2009), excessive ROS accumulation, also called oxidative stress, causes intramural damage and subsequent recruitment of inflammatory cells, increased lipid peroxidation, activation of matrix metalloproteases, and deposition of extracellular matrix. However, the molecular pathogenesis of ROS production and accumulation during hypertension-associated hemorrhagic stroke is unclear.

TRPA1 channels are present in the endothelium of cerebral arteries but aren’t found in endothelial cells from the mesenteric, coronary, renal, and dermal vascular beds (Sullivan et al., 2015). Endothelial cell TRPA1 channels dilate cerebral arteries and orchestrate neurovascular coupling in response to electrophilic compounds and ROS metabolites (Earley et al., 2009; Qian et al., 2013; Sullivan et al., 2015; Pires and Earley, 2018; Thakore et al., 2021). We have previously shown that ROS generated by the NOX2 enzyme indirectly activate TRPA1 channels in the cerebral artery endothelium by a pathway that requires the peroxidation of membrane phospholipids (Sullivan et al., 2015). TRPA1 channels activated in this manner produce subcellular Ca^2+^ signals that stimulate intermediate conductance Ca^2+^-activated K^+^ (IK) channels to evoke vasodilation (Sullivan et al., 2015). As NOX expression and ROS generation are enhanced in the vascular wall during hypertension (Rajagopalan et al., 1996; Zalba et al., 2001; Landmesser et al., 2003; Adler and Huang, 2004; Kazama et al., 2004; Paravicini et al., 2004; Matsuno et al., 2005), endothelial cell TRPA1 channel activity may also be elevated.

Here, we tested the hypothesis that increased ROS generation associated with severe hypertension increases endothelial cell TRPA1 activity to dilate cerebral arteries. We also investigated the role of TRPA1 during hypertension-associated hemorrhagic stroke. We induced hemorrhagic stroke by severely increasing blood pressure through combined angiotensin II (Ang II) administration, a high-salt (HS) diet *ad libitum*, and drinking water supplemented with the nitric oxide synthase (NOS) inhibitor Nitro-L-arginine methyl ester hydrochloride (L-NAME) in control (*Trpa1* ^fl/fl^) and endothelial cell-specific TRPA1 knockout (*Trpa1*-ecKO). All animals developed hypertension, and most that completed the protocol had intracerebral hemorrhages. Cerebral artery *Nox1*, *Nox2,* and *Nox4* expression and ROS generation capacity were enhanced during hypertension. TRPA1-dependent dilation of cerebral arteries in response to NOX activation was also elevated in hypertensive mice. Histological analysis revealed similar numbers of intracerebral hemorrhages in the brains of control and *Trpa1*-ecKO mice, but the lesions were significantly smaller in *Trpa1*-ecKO mice. However, overall morbidity and mortality didn’t differ between groups. Together, these findings suggest that enhanced ROS production during hypertension increases cerebral artery endothelial cell TRPA1 activity leading to vasodilation and expansion of hemorrhagic lesions.

## Results

### Endothelial cell TRPA1 knockout does not affect resting blood pressure or responses to hypertensive stimuli

**FIGURE 1:**
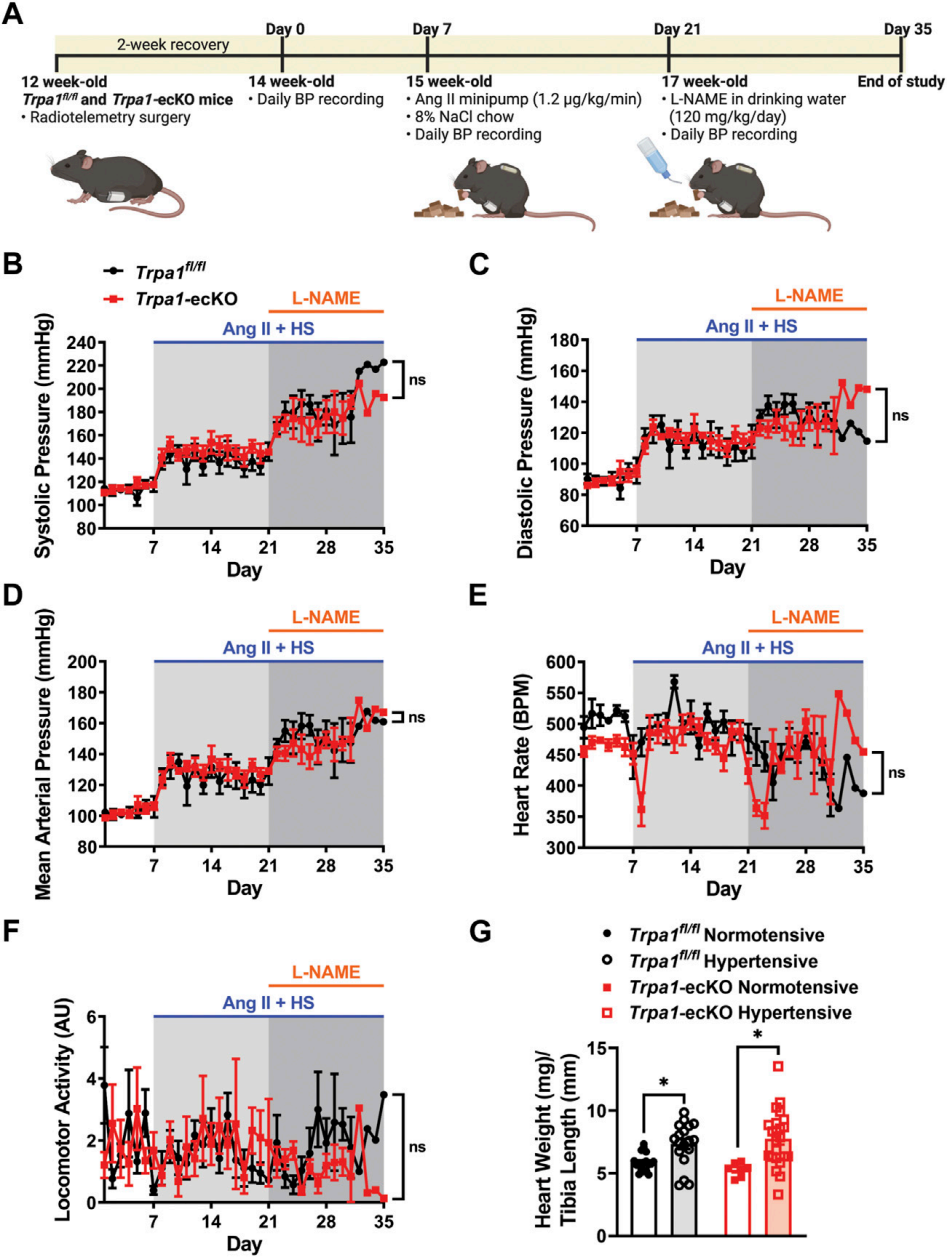
Endothelial cell *Trpa1* knockout does not affect resting BP or responses to hypertensive stimuli. **(A)** Schematic representation of the hypertension protocol. 12-week-old *Trpa1* ^*fl/fl*^ (control) and *Trpa1*-ecKO mice were surgically implanted with radiotelemetry probes to measure blood pressure (BP). Following a 2-week recovery, baseline BP was recorded for 7 days. These mice were then implanted with subcutaneous Ang II minipumps (1.2 μg/kg/min) and fed an 8% high-salt (HS) diet. After 2 weeks, the drinking water was supplemented with the NOS inhibitor L-NAME and treatment was continued for another 2 weeks. **(B–F)** Systolic BP **(B)**, diastolic BP **(C)**, mean arterial pressure **(D)**, heart rate **(E)**, and locomotor activity **(F)** were recorded from control and *Trpa1*-ecKO mice before and after Ang II/HS/L-NAME treatment (n = 8 for control, n = 6 for *Trpa1*-ecKO, two-way ANOVA, ns = not significant). **(G)** Heart weight normalized to tibia length was assessed from normotensive and hypertensive control and *Trpa1*-ecKO mice (n = 18 for normotensive control, n = 18 for hypertensive control, n = 7 for normotensive *Trpa1*-ecKO, n = 23 for hypertensive *Trpa1*-ecKO; two-way ANOVA, *p < 0.05).

Blood pressure (BP) and heart rate were measured in conscious, freely moving *Trpa1*-ecKO and control *Trpa1* ^fl/fl^ mice through surgical implantation of radiotelemetry transmitters as previously described (Feng et al., 2010; Li et al., 2014b; Thakore et al., 2020; Krishnan et al., 2022); mice were allowed to recover for 2 weeks. Animals were then chronically infused with Ang II (1.2 μg/kg/min) using osmotic minipumps, surgically inserted into the subcutaneous space, and fed a HS (8% NaCl) chow diet (Figure 1A). After 2 weeks of chronic Ang II/HS treatment, the NOS inhibitor L-NAME was introduced into the drinking water (120 mg/kg/day) to increase systemic BP further. Treatment was maintained for 28 days after minipump implantation, and BP, heart rate (HR), and locomotor activity were monitored daily (Figure 1A). Baseline systolic, diastolic, and mean arterial pressures, HR, and locomotor activity didn’t differ between *Trpa1* ^fl/fl^ and *Trpa1*-ecKO mice. Ang II/HS treatment increased systolic, diastolic, and mean arterial pressure in control and *Trpa1*-ecKO mice, and BP was further elevated after adding L-NAME to the drinking water (Figures 1B–D). These responses didn’t differ between *Trpa1* ^fl/fl^ and *Trpa1*-ecKO mice. The treatment didn’t alter HR or locomotor activity for either group (Figures 1E, F). Increased cardiac weight, suggestive of cardiac hypertrophy, was observed in *Trpa1* ^fl/fl^ and *Trpa1*-ecKO mice treated with Ang II/HS/L-NAME compared to their untreated counterparts (Figure 1G). However no differences between *Trpa1* ^fl/fl^ and *Trpa1*-ecKO mice were observed, suggesting that endothelial cell TRPA1 expression isn’t directly involved in cardiac hypertrophy associated with hypertension. These data indicate that Ang II/HS/L-NAME treatment induced severe hypertension and that genetic deletion of endothelial cell TRPA1 channels didn’t alter basal hemodynamics nor increases in BP in response to the hypertensive stimuli.

### ROS production and TRPA1-dependent cerebral artery dilation are enhanced during hypertension

**FIGURE 2:**
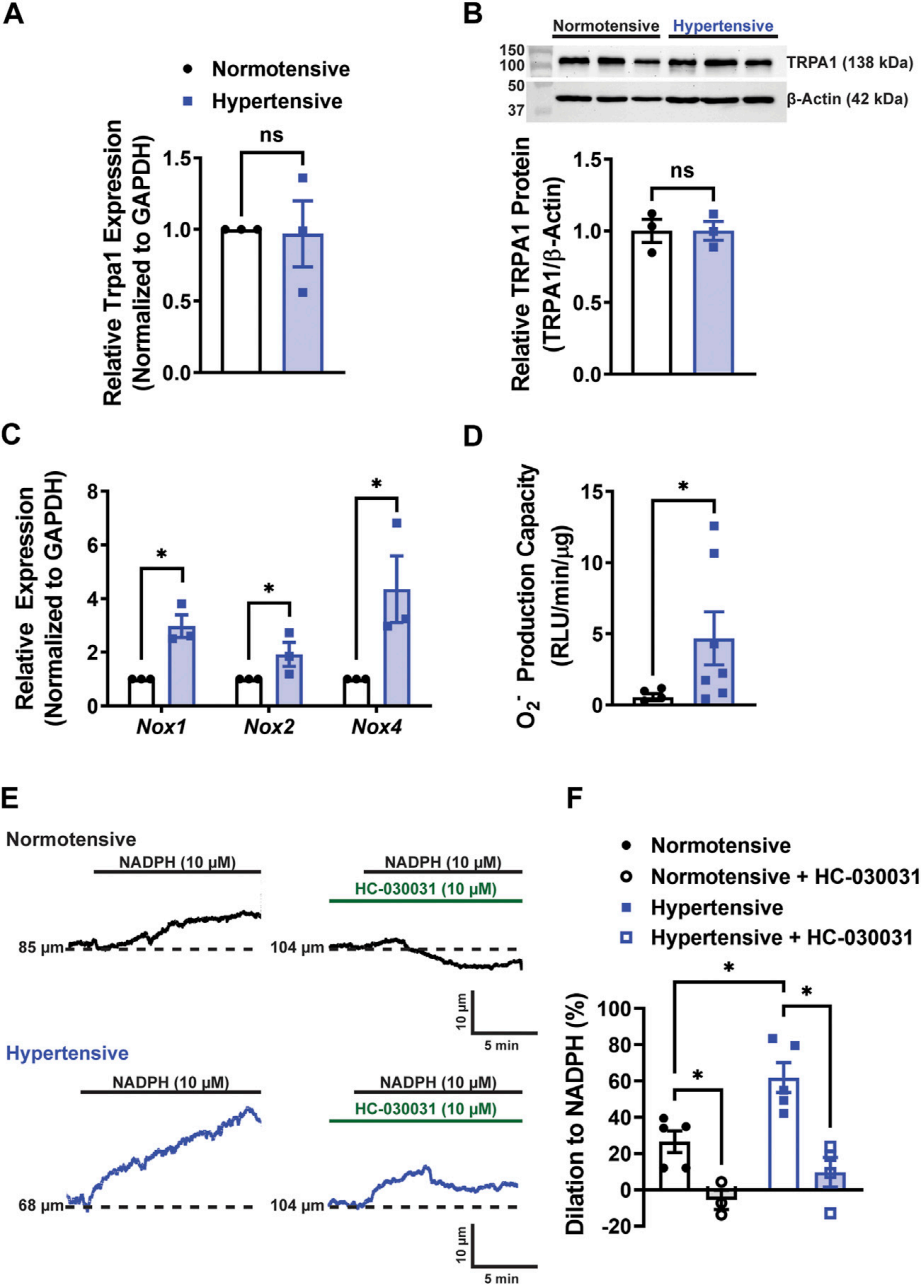
ROS production and TRPA1-dependent cerebral artery dilation are enhanced during hypertension. **(A)** Summary data showing relative *Trpa1* mRNA expression levels in cerebral arteries from normotensive and hypertensive *Trpa1* ^fl/fll^ mice (n = 3 per group, unpaired t-test, ns = not significant). **(B)** Representative Western blots and summary data showing relative TRPA1 protein expression levels in cerebral arteries of normotensive and hypertensive *Trpa1* ^fl/fll^ mice (n = 3 per group, unpaired t-test, ns = not significant). **(C)** Summary data showing the relative mRNA expression levels of *Nox1*, *Nox2,* and *Nox4* in cerebral arteries of normotensive and hypertensive *Trpa1* ^fl/fll^ mice (n = 3 per group, unpaired t-test, *p < 0.05). **(D)** Summary data showing the O_2_^−^ production as measured using a lucigenin assay in cerebral arteries of normotensive and hypertensive *Trpa1* ^fl/fll^ mice (n = 5 for normotensive, n = 7 for hypertensive, Mann-Whitney test, *p < 0.05). **(E)** Representative traces of changes in lumen diameter of isolated cerebral arteries from normotensive and hypertensive *Trpa1* ^fl/fll^ mice in response to NADPH (10 µM) before and after TRPA1 inhibition with HC-030031 (10 µM). **(F)** Summary data showing the vasodilation in response to NADPH in isolated cerebral arteries from normotensive and hypertensive *Trpa1* ^fl/fll^ mice, with and without the TRPA1 inhibitor HC-030031 (n = 5 for normotensive, n = 3 for normotensive + HC-030031, n = 5 for hypertensive, n = 3 for hypertensive + HC-030031, two-way ANOVA, *p < 0.05).

After the end of the treatment (28 days after minipump placement), a cohort of *Trpa1* ^fl/fl^ mice was euthanized, and cerebral arteries were harvested. *Trpa1* mRNA transcript (Figure 2A) and TRPA1 protein levels (Figure 2B) didn’t differ between normotensive and hypertensive animals. However, we found that mRNA levels of the ROS-producing enzymes *Nox1*, *Nox2,* and *Nox4* were enhanced in cerebral arteries from hypertensive mice (Figure 2C). Using a chemiluminescent detection method, we also found that the capacity for superoxide anion (O_2_^−^) production was elevated in cerebral arteries from hypertensive mice compared with normotensive animals (Figure 2D). We previously reported that administration of the NOX substrate NADPH dilated cerebral arteries by activating TRPA1 channels through lipid peroxidation (Sullivan et al., 2015). Here, we found that NADPH dilated cerebral arteries from hypertensive animals to a greater extent compared with normotensive mice (Figures 2E, F). The selective TRPA1 blocker HC-030031 attenuated NADPH-induced dilation (Figures 2E, F). These data suggest that the capacity for NOX-induced activation of TRPA1 channels and subsequent dilation of cerebral arteries are enhanced during hypertension.

### Endothelial cell TRPA1 knockout reduces the size of intracerebral hemorrhages during severe hypertension but does not impact outcomes

**FIGURE 3:**
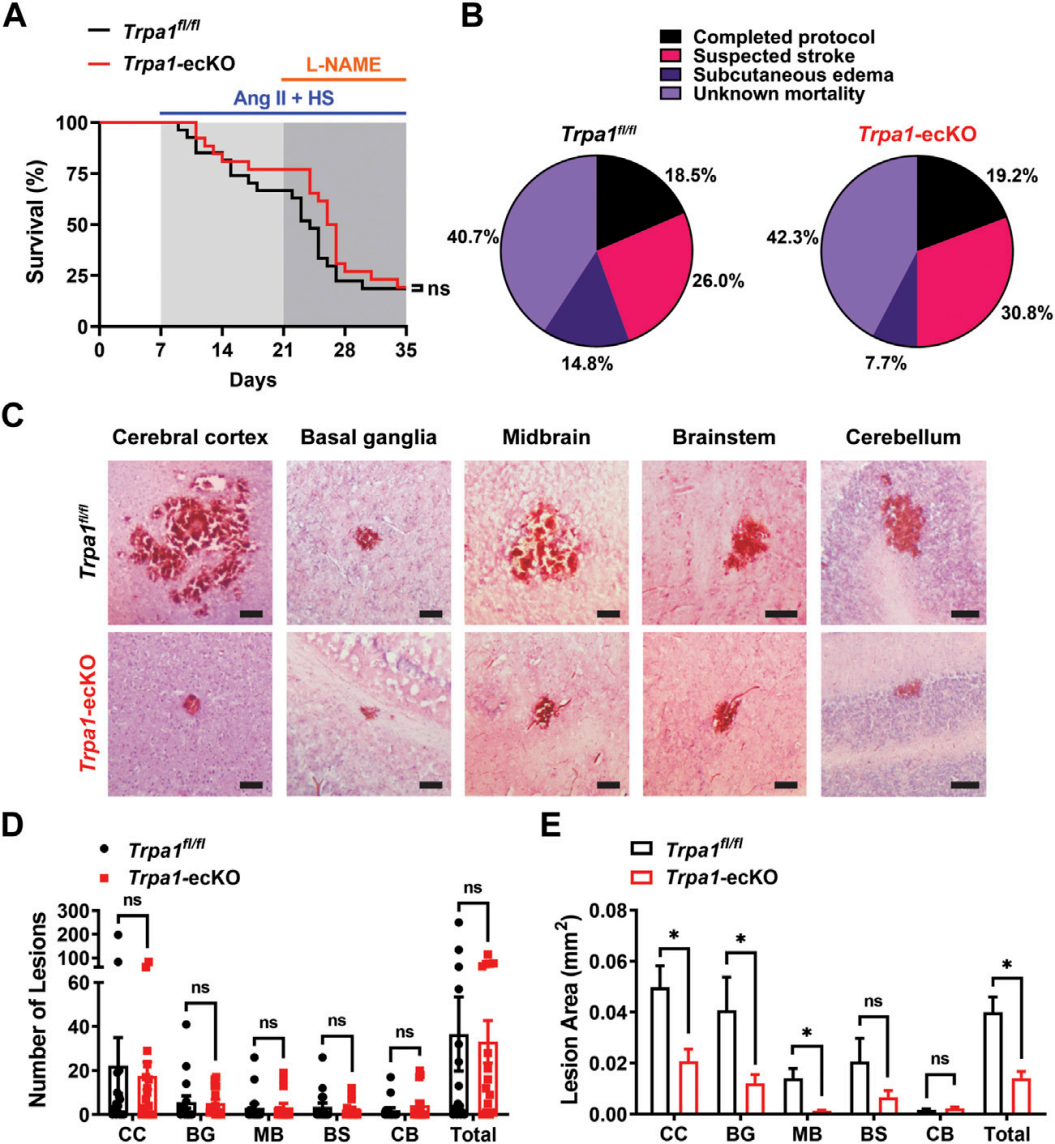
Endothelial cell *TRPA1* knockout reduces the size of intracerebral hemorrhages during severe hypertension but doesn’t improve other outcomes. **(A, B)** Kaplan-Meier plot **(A)** and pie charts indicating the distribution of outcomes **(B)** of *Trpa1* ^fl/fl^ and *Trpa1*-ecKO mice treated with Ang II/HS/L-NAME (n = 27 for control and n = 26 for *Trpa1*-ecKO, Log-rank (Mantel-Cox) test, ns = not significant). **(C)** Representative images of H and E stained brain sections from *Trpa1* ^fl/fll^ and *Trpa1*-ecKO mice treated with Ang II/HS/L-NAME. Lesions were found in the cerebral cortex (CC), basal ganglia (BG), midbrain (MB), brain stem (BS), and cerebellum (CB) in brains of mice from both groups. Scale bar = 100 µm. **(D)** Summary data showing the number of lesions per brain from *Trpa1* ^fl/fll^ and *Trpa1*-ecKO mice treated with Ang II/HS/L-NAME (n = 16 for control, n = 15 for *Trpa1*-ecKO, unpaired t-test, ns = not significant). **(E)** Summary data showing the lesion area in brains from *Trpa1* ^fl/fll^ and *Trpa1*-ecKO mice treated with Ang II/HS/L-NAME (CC: n = 357 for *Trpa1* ^fl/fll^ and n = 265 for *Trpa1*-ecKO; BG: n = 90 for *Trpa1* ^fl/fll^ and n = 79 for *Trpa1*-ecKO; MB: n = 50 for *Trpa1* ^fl/fll^ and n = 51 for *Trpa1*-ecKO; BS: n = 57 for *Trpa1* ^fl/fll^ and n = 36 for *Trpa1*-ecKO; CB: n = 31 for *Trpa1* ^fl/fll^ and n = 65 for *Trpa1*-ecKO; total: n = 585 for control and n = 496 for *Trpa1*-ecKO, unpaired t-test, *p < 0.05, ns = not significant).

Treatment with Ang II/HS/L-NAME was maintained for 28 days (after minipump implantation) or until mice exhibited signs of morbidity (hunched posture, squinted eyes, ruffled haircoat, dehydration, etc.), including clinical signs suggestive of stroke (circling behavior, head tilt, mobility impairment, muscular fasciculations, other motor dysfunction) (Iida et al., 2005) (Supplementary Videos S1, S2). Approximately 20% of mice from both groups completed the protocol without apparent morbidity (Figures 3A, B). Of the mice that didnt finish the study, about 40% were found deceased in the cage (mortality of unknown cause), 26%–31% displayed neurological dysfunction suggestive of stroke, and between 8% and 15% suffered from generalized, severe subcutaneous edema (Supplementary Figure S1) (Figure 3B). No differences in morbidities were observed between *Trpa*1^fl/fl^ and *Trpa1*-ecKO animals.

The volume of intracerebral hemorrhage lesions is commonly used as a prognostic indicator in stroke patients and is obtained using advanced diagnostic imaging methods, such as MRI (Broderick et al., 1993; Linfante et al., 1999; Kidwell et al., 2004). Due to the technical difficulties associated with high-resolution imaging of mouse brains *in vivo* and the acute onset of morbidity and mortality, lesion size was measured using a histological approach. Surviving mice and those removed from the protocol because of suspected stroke or subcutaneous edema were perfusion-fixed for histologic analysis. Their brains were removed, sectioned, and stained with hematoxylin and eosin (H and E) to identify sites of intracerebral hemorrhage (Figure 3C). Lesions were detected in 12 of 16 *Trpa*1^fl/fl^ mice and 13 of 15 *Trpa1*-ecKO animals (no significant difference between *Trpa*1^fl/fl^ and *Trpa1*-ecKO mice). Lesions were predominantly found in the cerebral cortex but were also detected in the basal ganglia, midbrain, brain stem, and cerebellum in brains of mice from both groups (Figures 3C and D). The number of lesions per brain did not differ between groups (Figure 3D), but the mean area of individual lesions was significantly smaller in the brains of *Trpa1*-ecKO mice compared to controls (Figure 3E). Lesions in the cerebral cortex, basal ganglia, and midbrain were significantly smaller in the brains of *Trpa1*-ecKO mice compared to control *Trpa*1^fl/fl^ mice. These data suggest that endothelial cell TRPA1 knockout reduces the size of intracerebral hemorrhages in hypertensive mice, but this effect doesn’t improve survival.

## Discussion

**FIGURE 4:**
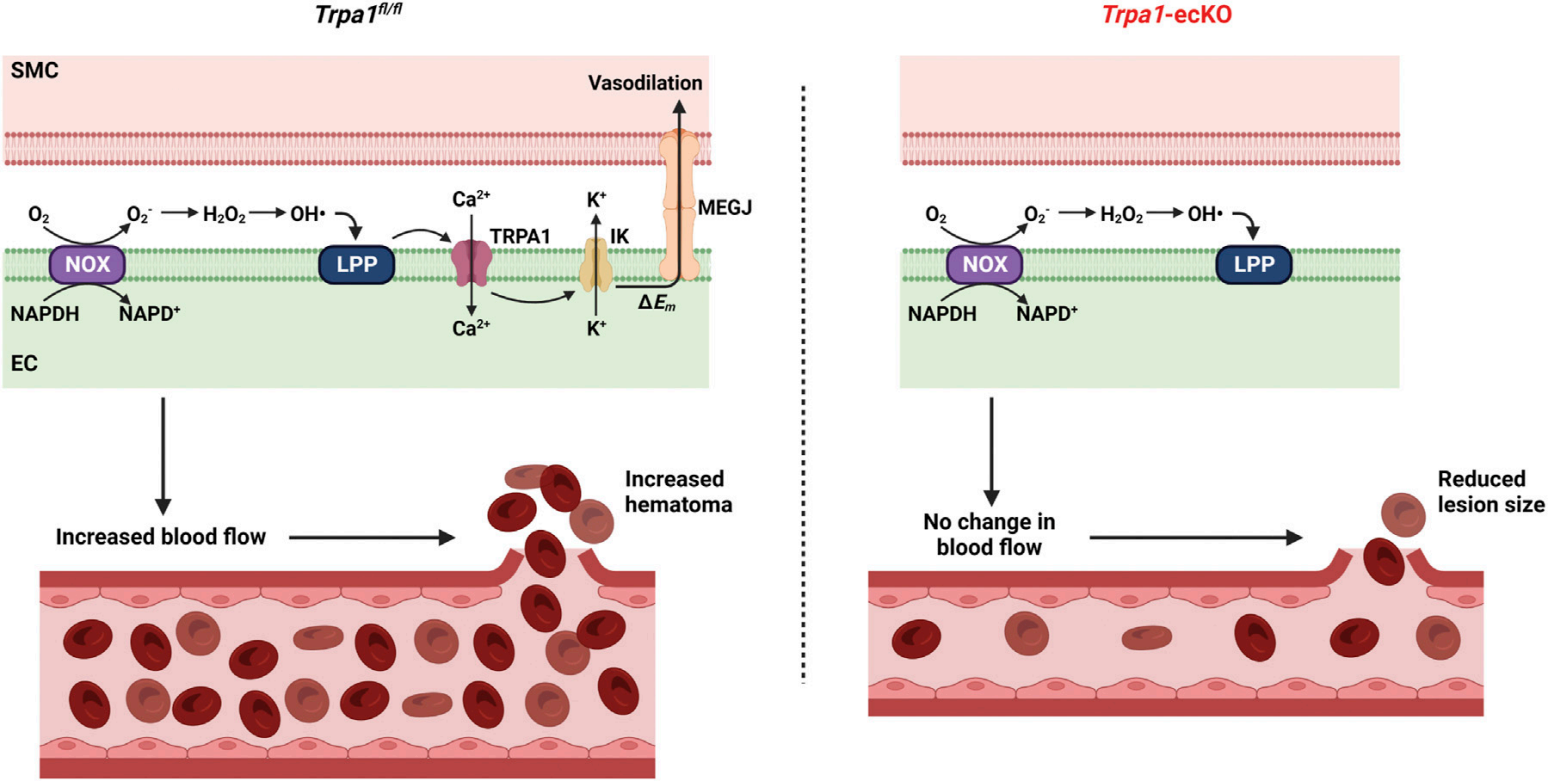
Hypothetical model of *TRPA1*-induced hematoma expansion*.* Severe hypertension increases NOX-dependent superoxide (O_2_^−^) generation in endothelial cells. O_2_^−^ is dismutased to hydrogen peroxide (H_2_O_2_), which undergoes the Fenton reaction to produce hydroxyl radicals (OH•). OH• oxidizes membrane lipids to generate lipid peroxidation products (LPP) which activate TRPA1 channels. Ca^2+^ influx through TRPA1 channels stimulates K^+^ efflux through intermediate conductance Ca^2+^-activated K^+^ (IK) channels leading to plasma membrane hyperpolarization (Δ*E*_*m*_). Hyperpolarization spreads to smooth muscle cells through myoendothelial gap junctions (MEGJ), resulting in vasodilation and increased blood flow, leading to a larger hematoma when vascular wall integrity is compromised. *Trpa1*-ecKO don’t experience ROS-induced vasodilation, resulting in smaller lesions.

The current study investigated the involvement of endothelial cell TRPA1 channels in the pathogenesis of hemorrhagic stroke associated with severe hypertension. Mice were subjected to a protocol that significantly increased systemic BP to levels that caused intracerebral hemorrhage in a majority of the animals studied. Induction of severe hypertension increased the capacity for ROS generation and TRPA1-dependent dilation of cerebral arteries by increasing the expression of NOX enzymes. Intracerebral hemorrhages were smaller in *Trpa1*-ecKO mice compared to controls, suggesting that TRPA1 channel activity-associated vasodilation and subsequent increase in cerebral blood flow may lead to hematoma expansion during intracerebral hemorrhage events (Figure 4). However, we didn’t observe any differences in survival or other outcomes between control and *Trpa1*-ecKO mice, suggesting that interventions targeting TRPA1 channels in the clinical setting may have minimal impact on hemorrhagic stroke patients.

Spontaneous hemorrhagic stroke is challenging to study in mice. The previously described models require daily, invasive interventions or are unwieldy to use with transgenic mice. For example, Wakisaka et al. (2010b) chronically infused mice with Ang II and supplied L-NAME in the drinking water, followed by twice daily subcutaneous injections of Ang II or norepinephrine to transiently increase blood pressure. This method increased systemic BP, but only the mice chronically infused and acutely injected with Ang II consistently developed stroke. Approximately 73% of mice injected with Ang II developed signs of stroke, compared to ∼40% of mice injected with norepinephrine (Wakisaka et al., 2010b). A study from Iida et al. (2005) described a genetic model of spontaneous hemorrhagic stroke using double transgenic mice overexpressing human renin and angiotensinogen (R^+^/A^+^) treated with an HS diet and L-NAME in the drinking water. R^+^/A^+^ mice are innately hypertensive but don’t spontaneously develop stroke. Upon treatment with HS and L-NAME, BP increases substantially, which correlates with the development of intracerebral hemorrhages in 100% of the mice. However, combining a double transgenic mouse line like R^+^/A^+^ mice with other transgenic lines (e.g., *Trpa1*-ecKO) requires extensive breeding to obtain animals with the four required transgenes. The Ang II/HS/L-NAME protocol consistently elevated BP in all mice can be readily applied to transgenic animals and doesn’t require daily procedures (e.g., injections). Approximately 81% of mice that completed this protocol developed hemorrhagic lesions, similar to the model reported by Wakisaka et al. However, about 40% of the animals undergoing this hypertensive treatment regimen died of unknown causes, somewhat limiting utility. The precise causes of death for these animals were unclear. It is possible that the animals may have had a fatal stroke, myocardial infarction, or aneurysm due to the severity of the blood pressure increase. However, these animals were found after rigor mortis had set in, making it difficult to perform the necessary investigations to identify the precise cause.

Hemorrhagic strokes cause higher rates of mortality and long-term disability in survivors compared with ischemic strokes (Virani et al., 2021). Brain damage secondary to intracerebral hemorrhage is multifactorial, including increased intracranial pressure due to hematoma formation, neuroinflammation, excitotoxicity, and subsequent edema and neuronal cell death (Xi et al., 2001). ROS produced by the breakdown of extravasated blood and the associated inflammatory response drive secondary brain damage after intracerebral hemorrhage. (Duan et al., 2016; Qu et al., 2016). In addition, expression of NOX1, NOX2, and NOX4 significantly increases in brain tissue and the cerebral vasculature after stroke, and these enzymes are a substantial source of pathological ROS following intracerebral hemorrhage (Wakisaka et al., 2008; Wakisaka et al., 2010a; Zhang et al., 2016). Genetic deletion or pharmacological inhibition of NOX2 and NOX4 reduces brain tissue damage and improves neurological outcomes following hemorrhagic stroke (Shin et al., 2002; Tang et al., 2005). Excessive ROS peroxidizes plasma membrane lipids and increases intracellular Ca^2+^ levels (Ermak and Davies, 2002; Van et al., 2016). Ca^2+^ overload triggers several downstream pathways that damage cells and organelles, ultimately leading to cell death (Eigel et al., 2004; Gu et al., 2011; Li et al., 2014a; Duan et al., 2016). However, the precise mechanisms downstream of NOX-generated ROS that drive Ca^2+^ influx in the context of hemorrhagic stroke are unknown. We have previously shown that ROS metabolites endogenously activate TRPA1 in cerebral artery endothelial cells to induce vasodilation (Sullivan et al., 2015; Pires and Earley, 2018). This mechanism was neuroprotective following ischemic stroke by redirecting blood to hypoxic areas of the brain, ensuring an adequate supply of oxygen and nutrients, and reducing overall infarct size (Pires and Earley, 2018). Interestingly, the current study shows that not only does this protection not extend to cases of hemorrhagic stroke, but instead, TRPA1 channel activity appears to exacerbate the size of the hemorrhagic lesions. We propose ROS-activated endothelial cell TRPA1 channels enhance cerebral blood flow, resulting in more profound blood extravasation during intracerebral hemorrhage events. Removal of this process by knocking out TRPA1 channels resulted in smaller hemorrhagic lesions within the parenchyma. A prior study reported that hematoma expansion is a significant factor for the acute progression of neuronal damage associated with intracerebral hemorrhages (Chen et al., 2014). Interestingly, although the hemorrhagic lesion area was significantly reduced in animals devoid of endothelial TRPA1 channels, we didn’t observe any differences in morbidity or mortality between control *Trpa1* ^*fl/fl*^ and *Trpa1*-ecKO mice. Thus, our data suggest that blocking TRPA1 channels may not be helpful for treating hypertension-associated hemorrhagic stroke.

## Materials and methods

### Animals

All animal procedures used in the study complied with the Guide for the Care and Use of Laboratory Animals and were approved by the Institutional Animal Care and Use Committees of the University of Nevada, Reno School of medicine, and Colorado State University. Endothelial cell-specific deletion of TRPA1 was achieved by initially crossing mice homozygous for loxP sequences flanking S5/S6 transmembrane domains of TRPA1 (floxed TRPA1; Strain number: 008,654; The Jackson Laboratory, Bar Harbor, Maine) with heterozygous *Tek* ^*Cre*^ mice which express *cre recombinase* (*cre*) driven by the promoter for the endothelial cell-specific receptor tyrosine kinase *TEK* (Strain number: 008,863; The Jackson Laboratory, Bar Harbor, Maine) to produce intermediate heterozygote mice which were used to generate *Trpa1-*ecKO mice, as previously described (Sullivan et al., 2015; Thakore et al., 2021). Mice homozygous for floxed TRPA1 and positive for *cre* were considered *Trpa1*-ecKO, and littermates homozygous for floxed TRPA1 but negative for *cre* were used as controls (*Trpa1* ^*fl/fl*^). Mice were kept on a 12-h light/dark cycle and were fed standard chow and regular water *ad libitum* unless otherwise specified. Our prior study demonstrated that TRPA1 expression is undetectable by Western blotting in cerebral arteries from *Trpa1-*ecKO mice (Sullivan et al., 2015).

### Radiotelemetry

At 12 weeks of age, mice underwent telemetry probe implantation as previously described (Feng et al., 2010; Li et al., 2014b; Thakore et al., 2020; Krishnan et al., 2022). Briefly, anesthesia was induced by 4%–5% isoflurane carried in 100% O_2_ (flow rate 1 L/min), after which anesthesia was maintained by adjusting isoflurane to 1.5%–2%; pre-operative analgesia was provided by a single subcutaneous injection of 0.05 mg/kg buprenorphine (Zoopharm, Windsor, CO). The neck was shaved and then sterilized with iodine. Under aseptic conditions, an incision (∼1 cm) was made to separate the oblique and tracheal muscles and expose the left common carotid artery. The catheter of a radio telemetry transmitter (PA-C10; Data Science International, Harvard Bioscience, Inc., Minneapolis, MN) was surgically implanted in the left common carotid artery and secured using non-absorbable silk suture threads. The body of the transmitter was placed in a subcutaneous pocket caudal to the right forelimb. After a 14-day recovery period, baseline BP, heart rate, and locomotor activity were recorded in conscious mice for 7 days. Data were collected daily every 10 s for 2 h (from 2 p.m. to 4 p.m.) for the duration of the study protocol and analyzed.

### Hypertension-induced spontaneous stroke

At 15 weeks of age, mice were surgically implanted with an osmotic minipump filled with Ang II (1.2 μg/kg/min; Sigma-Aldrich, St. Louis, MO) and fed 8% NaCl (HS; Envigo, Indianapolis, IN) chow *ad libitum*. After 2 weeks of Ang II and HS treatment, L-NAME (120 mg/kg/day; Sigma-Aldrich) was added to the drinking water. Treatment was continued for two additional weeks to induce severe hypertension and hemorrhagic strokes. Mice were monitored at least once daily. At the end of the treatment protocol, or sooner if mice showed behavioral signs of a stroke or other morbidity, mice were euthanized and perfusion-fixed in ice-cold 4% paraformaldehyde (PFA) solution for brain histology. Non-fixed brains were also collected from a cohort of *Trpa1* ^fl/fl^ mice for *ex vivo* analysis.

### Quantitative real-time RT-PCR

Cerebral arteries from three animals were isolated and pooled together from control normotensive and hypertensive animals. RNA was extracted and purified using a RNeasy mini kit (Qiagen, Hilden, Germany). A reverse transcriptase reaction was performed using 200 ng RNA per group with a QuantiTect RT kit (Qiagen) to generate complementary DNA (cDNA) samples. Quantitative PCR was performed using a QuantiTect SYBR Green kit (Qiagen). Primers for mouse *Gapdh* (QT01658692, Qiagen), *Trpa1* (QT01595937, Qiagen), *Nox1* (QT00140091, Qiagen)*, Nox2* (QT00139797, Qiagen)*,* and *Nox4* (QT00126042, Qiagen) were used to determine respective mRNA levels. This process was repeated twice to generate results from three independent biological samples. The relative expression levels were calculated using the Pfaffl method (Pfaffl, 2001) as previously described (Crnich et al., 2010)*.*

### Western blotting

Cerebral arteries were isolated from control normotensive and hypertensive animals and snap-frozen in liquid nitrogen. RIPA lysis buffer (50 µl) (Thermo Fisher Scientific, Waltham, MA) containing a protease inhibitor cocktail (Thermo Fisher Scientific) was added to each artery sample and homogenized by sonication (20 x 1-s pulses) and mechanical disruption by a Fisher Scientific Tissuemiser (10 s) on ice. Samples were centrifuged at 13,000 rpm for 10 min, and the supernatant was transferred to a new tube. Protein concentration for each sample was determined using a BCA Protein assay (Thermo Fisher Scientific). Protein samples (10 ng) were added to SDS sample buffer and heated at 70°C for 10 min. Immediately after denaturation, proteins were separated by SDS-PAGE and transferred to nitrocellulose membranes. Membranes were blocked with 5% milk and 1% BSA in PBS containing 0.1% Tween and 0.02% sodium azide (PBS-TA) for 30 min at room temperature on a rocker and then exposed to a rabbit anti-TRPA1 antibody (1:500, ACC-037 ***,*** Alomone Labs, Jerusalem, Israel) in 5% milk, 1% BSA (PBS-TA) overnight at room temperature on a rocker. The same blot was probed independently with a rabbit β-actin antibody (1:1,000, ab8227 ***,*** Abcam, Cambridge, United Kingdom). The membranes were then washed with PBS-T for 3 × 5 min and exposed to a goat anti-rabbit secondary antibody (1:10,000, Invitrogen) in 5% milk, 1% BSA (PBS-T) for 2 h at room temperature on a rocker, followed by 5-min washes with PBS-T, incubated in Supersignal ECL substrate (Thermo Fisher Scientific) for 1–3 min, and imaged. Protein bands were quantified using ImageJ software (version 1.52n, National Institutes of Health, Bethesda, MD).

### Lucigenin assay

Cerebral arteries were isolated from control normotensive and hypertensive animals. RIPA lysis buffer (50 µL) containing a protease inhibitor cocktail was added to each artery sample and homogenized by sonication (20 × 1-s pulses) and mechanical disruption by a Fisher Scientific Tissuemiser (10 s) on ice. Samples were centrifuged at 13,000 rpm for 10 min, and the supernatant was transferred to a new tube. Protein concentration for each sample was determined using a BCA Protein assay (Thermo Fisher Scientific). The isolated protein (∼50–60 µl) was diluted to 400 µL with HEPES-buffered saline, and lucigenin reagent (5 μM; Cayman Chemicals, Ann Arbor, MI) was added. At this concentration lucigenin doesn’t produce excess superoxide and provides an accurate assessment of the rate of superoxide production (Skatchkov et al., 1999; Munzel et al., 2002; Guzik and Channon, 2005). 200 µL was added to each of two wells in a 96-well plate. Apocynin (100 µM) was added to one of the wells, and the other was treated with vehicle (DMSO). Basal luminescence was measured in relative light units (RLU) every minute for 10 min using a BioTek Synergy H1 microplate reader (Biotek, Winooski, VT). NADPH (200 μM, Sigma-Aldrich) was then dispensed into each well, and luminescence was measured every minute for 50 min. O_2_^−^ production was calculated as the change in RLU/minute per µg protein. This *ex vivo* assay measures the capacity for O_2_^−^ production, but not O_2_^−^ level in cerebral arteries *in vivo*.

### Pressure myography

Isolated vessel experiments were performed as previously described (Sullivan et al., 2015; Wenceslau et al., 2021). Briefly, mouse middle cerebral arteries were isolated and placed in ice-cold MOPS-buffered saline. Arterial segments were then cannulated between two glass cannulas in a chamber (Living Systems Instrumentation, St. Albans City, VT) and secured with single-filament silk. Arteries were superfused with a physiological saline solution (PSS; 119 mM NaCl, 4.7 mM KCl, 21 mM NaHCO_3_, 1.17 mM MgSO_4_, 1.8 mM CaCl_2_, 1.18 mM KH_2_PO_4_, 5 mM glucose, 0.03 mM EDTA) warmed to 37°C and bubbled with 21% O_2_, 6% CO_2_, and balanced N_2_. Vessels were pressurized to 60 mmHg and gently stretched to simulate physiological conditions. Intraluminal pressure was lowered 20 mmHg and arteries were equilibrated for 15 min. Viability was assessed by superfusing a high [K^+^] PSS (60 mM KCl, 63.7 mM NaCl) to induce vasoconstriction. Follow a 15 min wash, intraluminal pressure was increased to 60 mmHg, and arteries were allowed to develop spontaneous myogenic tone. The % dilation was calculated as the % change in myogenic tone before and after the addition of NADPH.

### Histology

Brains were removed from perfusion-fixed mice and stored overnight in 4% PFA. Brains were then serially dehydrated in 7.5%, 15%, and 30% sucrose (Sigma-Aldrich) solutions at 4°C and frozen in OCT sectioning medium at −80°C. The tissue was sectioned with a cryostat (Leica Biosystems, Wetzlar, Germany), generating 35 µm thick sections collected every 500 µm. Sections were mounted on glass slides (Superfrost Plus, VWR International, Radnor, PA) and refrigerated overnight. These sections were then stained with hematoxylin and eosin using the H&E staining kit (Abcam, Cambridge, United Kingdom), per the manufacturer’s instructions. Stained sections were sealed using the Cytoseal XYL mounting medium (Thermo Fisher Scientific) and allowed to dry for a minimum of 24 h. Sections were imaged using a bright field microscope (BZ-X710, Keyence Corporation, Osaka, Japan) at ×4 magnification.

Intracerebral hemorrhage lesions were identified as an abnormal appearance of blood within the brain tissue (appears red by eosin staining). Serial coronal sections of brains were viewed by light microscopy, and the number of hemorrhagic lesions within each of the following brain regions was recorded: cerebral cortex, basal ganglia, midbrain, brainstem, or cerebellum. ImageJ image analysis software was used to measure each lesion’s maximum area (mm^2^).

### Statistical analysis

All summary data are presented as means ± SEM. Statistical analyses and graphical presentations were performed using GraphPad Prism software (version 9.5.0, GraphPad Software, San Diego, CA). The value of *n* refers to the number of mice per group used for radiotelemetry experiments, survival assays, lesion number quantification, qRT-PCR and Western blots. For lesion area quantification, *n* refers to the number of lesions from each mouse, and for isolated vessel experiments, *n* is the number of arteries per group. Statistical analyses were performed using Students paired or unpaired two-tailed t-test with or without Welch’s correction as appropriate, repeated measures or non-repeated measures two-way analysis of variance (ANOVA) with a Šidák correction for multiple comparisons, or Log-rank (Mantel-Cox) test. A value of *p* < 0.05 was considered as statistically significant.

## Data Availability

The original contributions presented in the study are included in the article/Supplementary Material, further inquiries can be directed to the corresponding author.
