# Supplementary figures and images for: Endothelial cell TRPA1 activity exacerbates cerebral hemorrhage during severe hypertension
Source: Front Mol Biosci. 2023 Jan 30;10:1129435. doi: 10.3389/fmolb.2023.1129435 (PMC9922848; doi:10.3389/fmolb.2023.1129435)

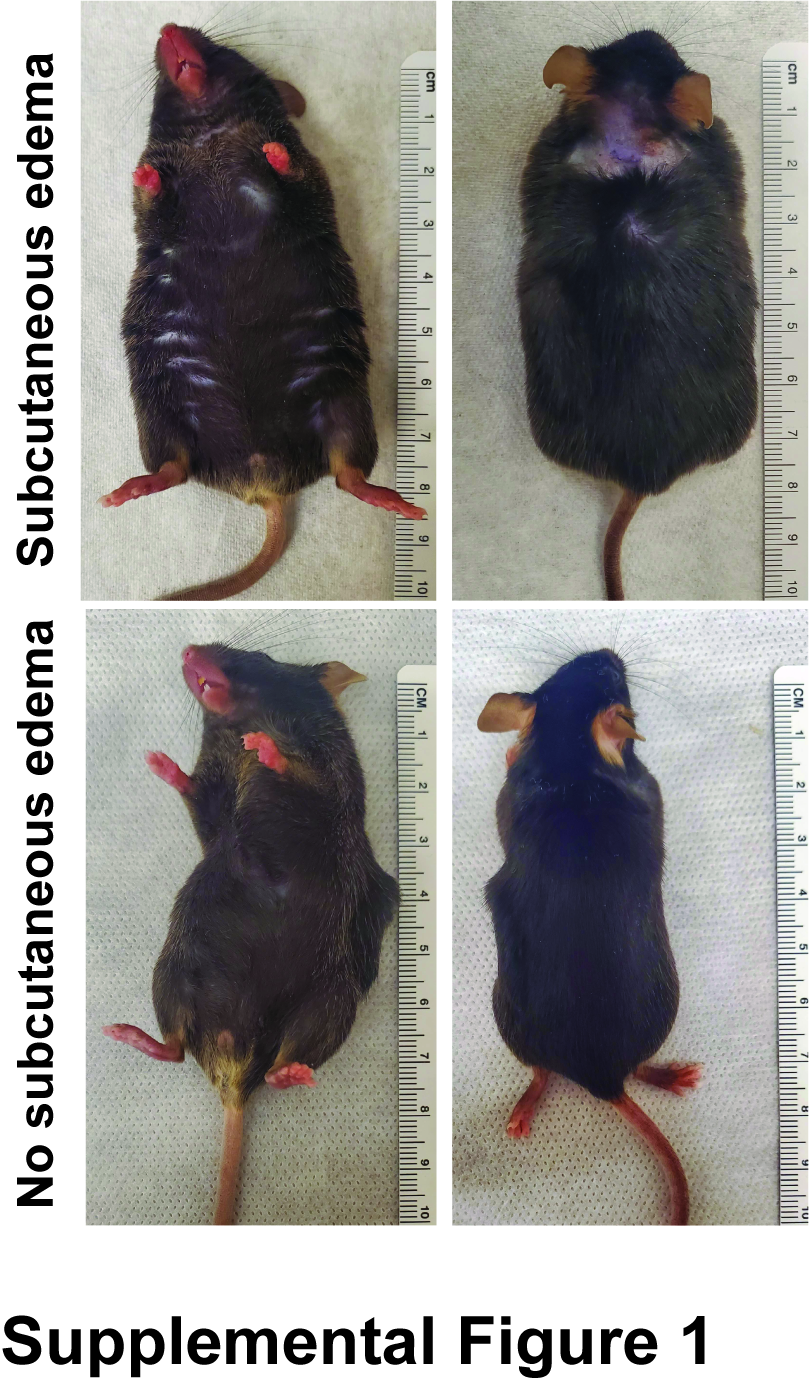

Supplement: Supplementary file 1 [file Image1.TIF]
